# Gold Carbide: A Predicted Nanotube Candidate from First Principle

**DOI:** 10.3390/nano11123182

**Published:** 2021-11-24

**Authors:** Xiaohang Lin, Lin Song, Anchen Shao, Minghao Hua, Xuelei Tian

**Affiliations:** Key Laboratory for Liquid-Solid Structural Evolution and Processing of Materials, Ministry of Education, Shandong University, Jinan 250061, China; songlinwork@outlook.com (L.S.); sacboyvkx@hotmail.com (A.S.); huaminghao@outlook.com (M.H.); tianxuelei@sdu.edu.cn (X.T.)

**Keywords:** AuC films, DFT calculations, folding effect, nanotube structure, electronic structure

## Abstract

In the present work, density functional theory (DFT) calculations were applied to confirm that the gold carbide previously experimentally synthesized was AuC film. A crucial finding is that these kinds of AuC films are self-folded on the graphite substrate, leading to the formation of a semi-nanotube structure, which significantly diminishes the error between the experimental and simulated lattice constant. The unique characteristic, the spontaneous archlike reconstruction, makes AuC a possible candidate for self-assembled nanotubes. The band structure indicated, in the designed AuC nanotube, a narrow gap semiconductor with a bandgap of 0.14 eV. Both AIMD (at 300 and 450 K) results and phonon spectra showed a rather high stability for the AuC nanotube because a strong chemical bond formed between the Au–5d and C–2p states. The AuC nanotube could become a novel functional material.

## 1. Introduction

Nanotubes, especially carbon nanotubes (CNTs), have attracted much attention because of their specific surface area, large aspect ratio, and excellent mechanical strength [1,2,3,4]. The tensile strength of CNTs is 100 times larger than that of steel, and the electrical/thermal conductivity is close to that of copper [5,6]. These unique properties make nanotubes good fillers in different polymers and ceramics, and are widely applied in various fields [7,8]. CNTs could also replace silicon-based transistors [9]. However, CNTs alone are far from meeting diverse needs. In order to investigate the specific structure (nanotube) and special properties derived from it, researchers keep searching for new candidates.

In 1900, explosive gold acetylide Au_2_C_2_ was reported by Mathews and Watters [10]. Gold carbides (AuC), mostly AuC nanoclusters, are widely studied in the organic gold chemistry field and have potential applications in molecular electronics, luminescence, molecular recognition, optical switch, and catalysis direction [11,12,13,14,15,16]. However, compared with other pure metal carbides, there are hardly any relevant reports on stable inorganic crystalline gold carbon compounds and their specific structural properties under experimental conditions [17]. This could be explained well by the fact that gold and carbon are almost completely immiscible in thermodynamic equilibrium. The experimental synthesis of gold carbide clusters is also unstable and easy to decompose at room temperature. Moreover, these structures are usually charged, which makes the synthesized clusters very small [18].

In our previous work, the in situ heating method of electrically biased graphene was applied to the interaction between Au nanoislands and a graphene substrate [18]. The bottom-up formation of a novel and stable material was observed by aberration-corrected high-resolution transmission electron microscopy (AC-HRTEM). The robust structure can also be proved from the side by the experiment where a relatively strong Au–graphene interaction was observed under high temperature [19]. The lattice constant of the new material was 4.75 Å, which was confirmed as a AuC crystal structure by density functional theory (DFT).

However, limited by experimental conditions (small amounts and impurities), the microstructure and properties of gold carbon compound nanoparticles are not yet clear, which greatly hinders the development of this novel material. Considering the difficulty and progress of the experimental synthesis of gold carbide, DFT simulation allows for investigation of the structure and properties of AuC crystals, surfaces, and nanoparticles, including the electronic structure, and physical and chemical properties [20,21,22,23,24,25].

In the present work, the properties of AuC surfaces and thin films were investigated by DFT calculation. The phenomena that AuC nanoparticles cause automatically indicate a new potential self-assembly nanotube material. In addition, ab initio molecular dynamics (AIMD) simulations were performed at temperatures of 300 and 450 K, in order to assess the thermal stability. DFT and AIMD simulations of the AuC nanotube showed that it is a stable 1D semiconductor material with a narrow gap, which indicates that it is a potential nanotube candidate.

## 2. Methods

In this work, all DFT total energy calculations were performed by with the Vienna ab initio simulation package (VASP6.1) (VASP Software GmbH Sensengasse 8/12 A-1090, Vienna, Austria) within the generalized gradient approximation (GGA) to describe the exchange-correlation effects. The Perdew, Burke, and Ernzerhof (PBE) exchange-correlation functional [26,27] was used to describe the system without graphite [28,29,30,31], which was carefully checked to confirm that the van Der Waals interaction will not significantly change the structure (difference less than 0.8%). The PBE-D3 functional was used to describe the AuC–graphite systems. Structures were optimized until atomic forces had converged to 0.01 eV/Å. The plane wave cut-off energy was set to 500 eV. Different surface unit cell sizes were employed in these studies. In each case, special k-points sets were used to replace the integration over the first Brillouin zone, and were chosen to be large enough to yield converged results.

A typical two-sided slab model constructed by selectively exposing specific planes and removing some atoms to form a vacuum was proposed to simulate the surface. Compared with pure elemental materials, the surfaces of compounds are more complex, as the stoichiometry at the surfaces might be different from the one in bulk. For nonstoichiometric surfaces, the gas-phase environment contributes to surface stability through corresponding chemical potentials. In this case, the chemical potential of the constituents should be considered. The surface energy γ of a certain compound surface is given by

(1) $$\gamma =\frac{1}{A}\left[E_{tot}-\mu_{AuC}\times N_{C}-\mu_{Au}\times \left(N_{Au}-N_{C}\right)\right]$$

where *A* is the surface area, $E_{tot}$ is the total energy of the surface per unit cell, and $\mu_{AuC}$ and $\mu_{Au}$ are the chemical potential of AuC compounds and pure Au metal, respectively. $N_{Au}$ and $N_{C}$ are the number of Au and C atoms per 1 × 1 AuC surface unit cell. Stoichiometry $\Delta N=N_{Au}-N_{C}$ simply gives the slope of the surface energy with respect to the chemical potential [32].

In fact, most AuC surfaces that we consider are not stochiometric. However, the different surface energies for asymmetric slabs cannot typically be separated, so that only an average surface energy can be derived. In the case of the AuC surfaces, we could always construct slabs with the same first layer of atoms, but the second layer could differ. We take the average value, which is reasonable considering that the first layer on both sides is the same. Therefore, bulk energy cannot be used as the only reference for the surface energy.

In addition, chemical potential is related to the elemental bulk chemical potentials through the heat of formation ∆*H_AuC_*. Therefore, we obtained the possible range of Au chemical potentials that is determined by the energy of bulk Au and the heat of formation, which is given by following formula:

(2) $$\mu_{Au}^{bulk}-\Delta H_{AuC}<\mu_{Au}<\mu_{Au}^{bulk}$$

AIMD simulations were performed within the microcanonical ensemble using the Verlet algorithm for 8 ps with a time step of 1 fs (8000 steps). Thermal runs were carried out at temperatures of 300 and 450 K, starting with the optimized AuC nanotube geometries.

## 3. Results and Discussions

In previous studies, the lattice constants of AuC (ZnS structure) were calculated by DFT simulation. A certain deviation between experiment and simulation was found, in which the DFT calculation result (4.91 Å) was about 3.37% larger than the experimental value (4.75 Å) [18]. Even if considering the measurement error, this difference was still relatively large for a structural simulation using a DFT code [33,34,35]. The relatively large error can be explained by the fact that the AuC synthesized in the experiment was strictly a thin film nanoparticle rather than a crystal as studied by previous DFT calculations. Therefore, the special properties of the AuC surface and nanoparticles were investigated, including surface character, electronic structure, chemical potential, and mechanical properties, in order to deeply understand the experimental phenomenon.

**Figure 1:**
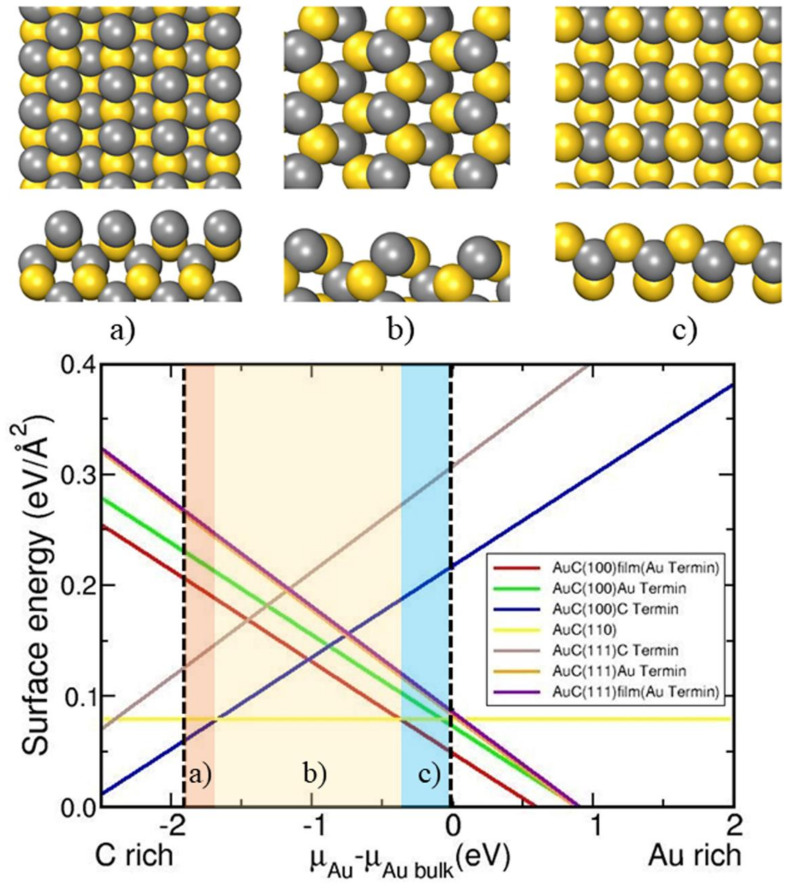
Surface energies of different AuC surfaces in eV/Å as function of Au chemical potential with respect to bulk Au. Perpendicular dashed lines indicate range of possible Au chemical potentials. Thermodynamically stable phases in different chemical potential area marked in red, yellow, and blue: (**a**) AuC (100) C-terminated surface, (**b**) AuC (110) surface, and (**c**) AuC (100) Au-terminated film, respectively.

**Figure 2:**
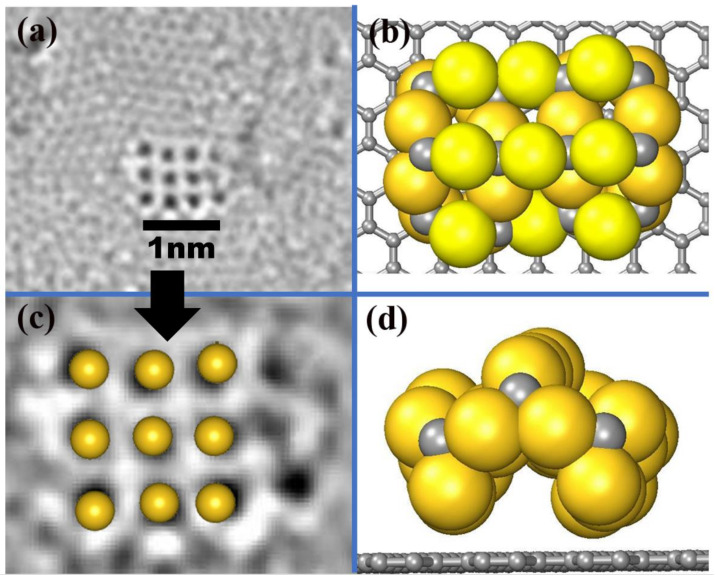
(**a**) AC-HRTEM images at 80 keV showing atomic arrangements of gold atoms adsorbed on graphite substrate [8]. (**b**) Top and (**d**) side views of AuC nanoparticle on graphite. Gold and grey balls represent gold and carbon atoms. (**c**) Comparison between HRTEM image and uppermost Au atoms of simulated AuC nanoparticle.

As the first step, AuC surfaces were calculated. Figure 1 plots the surface energies as a function of the chemical potential of the constituents according to Equation (1). There, the dashed lines display the range of possible Au chemical potentials given by Equation (2), with ∆*H_AuC_* = 1.03 eV obtained from our calculation. Low Au chemical potential corresponds to a C rich environment, while the Au bulk chemical potential gives the Au-rich limit. The surface with the lowest surface energy γ corresponds to the thermodynamically stable phase at a certain chemical potential. The most stable AuC surfaces were the AuC (100) C-terminated surface (C-rich condition; Figure 1a), AuC (110) surface (Figure 1b), and AuC (100) Au-terminated film (Au-rich condition; Figure 1c). Films with different thicknesses were calculated (from 3 to 21 layers), and the one with the lowest γ (film with three layers) was plotted in the phase diagram. The experimental results showed that the AuC nanoparticles were always adjacent to the Au island, in which the AuC (100) film that is gold terminated should be the most stable phase. This result agrees with that of the HRTEM experiment, in which nanoparticles with (100) structures were observed (Figure 2a). In order to compare with the experimental data, the film structure with three layers (Au terminated) was considered to be the nanoparticle structure in the following investigation.

In order to clearly understand the behavior of the AuC nanoparticle and directly compare it with the high-resolution transmission electron microscopy (HRTEM) (FEI NanoPorts, Hillsboro, USA) image, a 3 × 3 AuC nanoparticle with the most stable structure (AuC (100) Au-terminated film under Au-rich condition) adsorbed on graphite was simulated. A reconstruction spontaneously occurred. The structure bent up at the middle site, which produced an archlike structure (Figure 2b,d). The distance between two neighboring Au atoms was 0.24 Å smaller than the one on the AuC (100) surface. In this case, the lattice constant of the nanoparticle was only about 1% smaller than the experimental one. Although the nanoparticle was not flat, the positions of the uppermost gold atoms almost perfectly matched the HRTEM image (Figure 2c). This indicates that the structural model for AuC nanoparticles on graphite illustrated in Figure 2b is correct.

**Figure 3:**
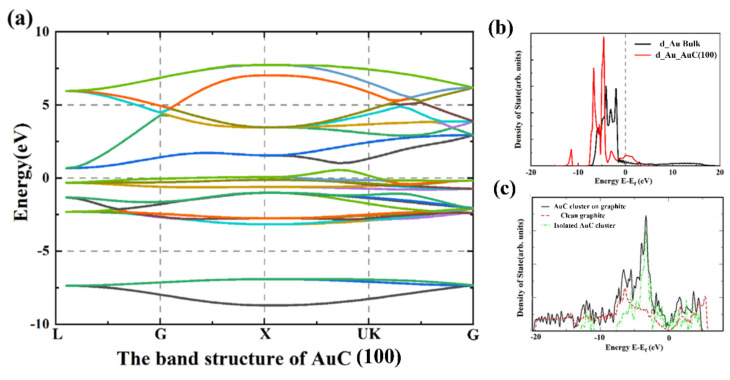
Electronic properties of AuC (100) film. (**a**) Band structure of AuC (100) film; (**b**) d-DOS of Au atom of Au bulk (black line) and AuC (100) film (red line); (**c**) DOS of clean graphite (red dashed line) and AuC nanoparticle in absence (green line) and presence (black line) of graphite substrate.

To deeply understand the electronic properties of the AuC nanoparticle, the band structure and local electron density of state (LDOS) were calculated as shown in Figure 3. The band structure showed that the AuC (100) film was a typical conductor, and the state density was rather low, indicating a relatively low conductivity of the AuC (100) film. Compared with the Au atom from the gold bulk, the LDOS of the Au atom d electron from the AuC (100) film split into two peaks located on the left and right sides of the Fermi level, which indicates a relatively strong Au–C interaction. Details of the Au–C interaction are discussed below.

In order to clarify the influence of the graphite substrate, the AuC nanoparticle without substrate was also calculated. The structure also spontaneously transferred into an archlike structure, as that in Figure 2d, which indicates that the reconstruction of the AuC nanoparticle should have the properties of the AuC film. Furthermore, this indicates a weak adsorbate–substrate interaction. Figure 3c shows the electron density of state (DOS) of AuC with or without graphite and clean graphite. The DOS of AuC on graphite did not significantly differ with the combination of isolated AuC nanoparticle and clean graphite. Because of the relatively weak interaction between the substrate and adsorbate, the total DOS was hardly influenced by the presence of the graphite substrate. The archlike structure of the AuC nanoparticle was mainly caused by its own characteristics. This can also be proven by the fact that the Au–C bond in the reconstructed structure was shorter and stronger.

**Figure 4:**
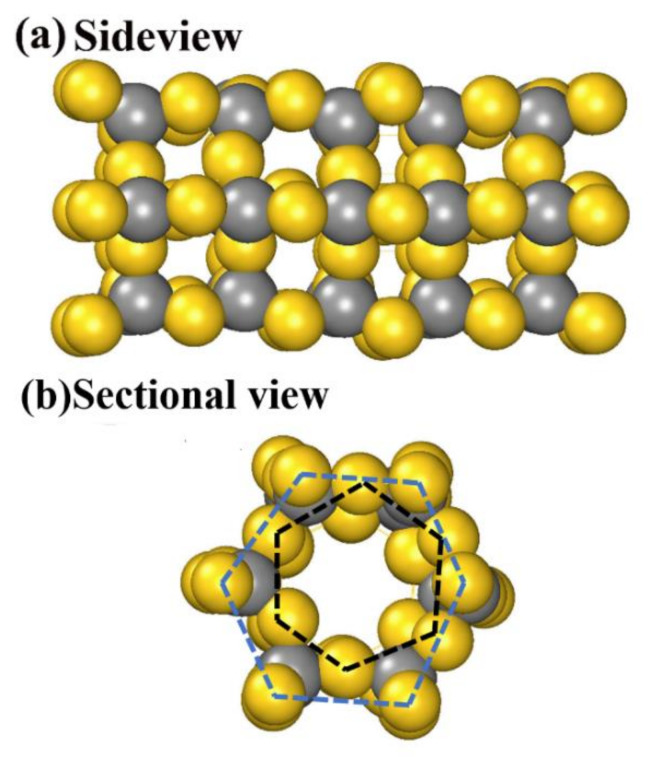
AuC nanotube structure: (**a**) side and (**b**) sectional views. Gold and grey balls represent gold and carbon atoms, respectively.

The unique characteristic of AuC, the spontaneous archlike reconstruction, makes it a possible candidate for self-assembled nanotubes. A designed nanotube based on the archlike structure in Figure 2b,d was calculated and is shown in Figure 4. First, the AuC nanotube showed high symmetry and kept the tubelike structure. The nanotube was formed by two Au hexagon rings marked by the black and blue dashed lines (Figure 4b), which were combined by six carbon atoms. The internal diameter was 5.80 Å, and the external diameter was 10.06 Å. Every gold atom was bonded with two carbon atoms, while every carbon atom was bonded with four gold atoms. The Au–C bond length was about 2.05 Å, which was 0.08 Å smaller than that in bulk, which also indicated a stronger Au–C interaction. The structure period was about 4.2 Å. Nanotubes with a different diameter could also exist, and we aim to investigate them. In this research, only the structure that might self-assemble into nanotubes was studied, according to Figure 2b,d.

**Figure 5:**
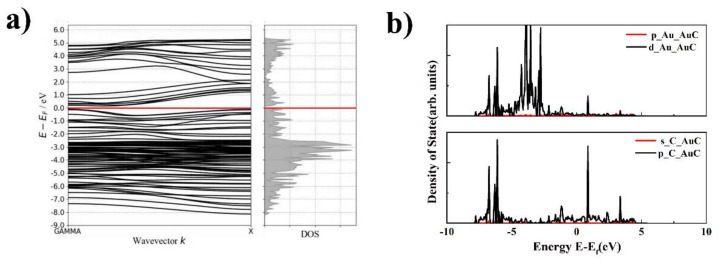
Band structure and electronic structure density of states (DOS) of AuC nanotube. (**a**) band structure of AuC tube; (**b**) DOS of AuC nanotube upper figure: p and d orbitals of Au in AuC nanotubes, lower figure: s and p orbitals of C in AuC nanotubes.

The electronic properties, including the band structure and LDOS of the AuC nanotube, were studied to further understand this novel nanotube, which is shown in Figure 5. The band structure indicated that the AuC nanotube was a narrow gap semiconductor with a bandgap of 0.14 eV. Although the DFT simulation using the GGA exchange-correlation functional always underestimates the bandgap, this does not change the fact that the AuC nanotube was a semiconductor. This change gives the AuC nanotube a greater potential for use in electronic devices. The archlike reconstruction was the main reason that the band structure of the AuC nanotube changed, which might be a way to regulate the band structure by changing the nanotube diameter. The LDOS of the AuC nanotube (Figure 5b) shows that the LDOS of the Au 5d electron split even more significantly compared with that of the AuC (100) film. The Au–5d and the C–2p states both exhibited peaks at around −1.1 and 0.9 eV, indicating a strong hybridization between them, and the formation of bonding and antibonding states resulting from the hybridization. The bonding contribution was fully occupied, and the antibonding contribution was unoccupied, which indicated that a strong chemical bond was formed between these two states.

**Figure 6:**
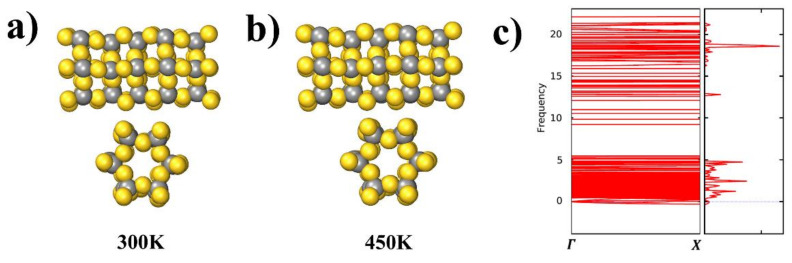
(**a**) and (**b**) Snapshots of AIMD simulations of AuC nanotube side and sectional views at 300 and 450 K after 8 ps; (**c**) phonon spectrum of AuC nanotubes with frequency in THz.

To assess the stability of the AuC nanotube at finite temperatures, AIMD simulations at 300 and 450 K were performed for a run time of 8 ps. Snapshots of the AIMD results after 8 ps at both 300 and 450 K are shown in Figure 6a. The AuC nanotube structure hardly changed its structure at room temperature, and even at 450 K, there was no indication of any structural rearrangement, which exhibited a rather high stability.

The calculated phonon structure for the AuC nanotube and selected high-symmetry directions (Γ–X in one-dimensional tube) in the BZ are shown in Figure 6b. The phonon spectra consisted of two regions separated by a wide gap (about 5 THz), which was mainly caused by the large mass difference between Au and C atoms leading to the splitting of cation and anion vibrations. The phonon dispersions of the AuC nanotube showed almost no imaginary modes (less than 0.01 THz), suggesting that this compound is dynamically stable.

## 4. Conclusions

In the present work, DFT calculations were applied to confirm that the previously experimentally synthesized gold carbide was AuC film. A self-folding reconstruction of the AuC films occurred on the graphite substrate, which significantly diminished the error between the experimental and simulated lattice constants. The unique characteristic, the spontaneous archlike reconstruction, makes AuC a possible candidate of self-assembled nanotubes. The band structure indicated that the designed AuC nanotube was a narrow gap semiconductor with a bandgap of 0.14 eV. Both AIMD (at 300 and 450 K) results and phonon spectra showed the rather high stability of the AuC nanotube, which can be explained by the strong chemical bond that formed between the Au–5d and C–2p states. The AuC nanotube could become a novel functional material.
